# Minimally Invasive Bone Regeneration in Implant Dentistry: From Biological Principles to Indication-Driven Clinical Decision-Making—A Narrative Review

**DOI:** 10.3390/jcm15114208

**Published:** 2026-05-29

**Authors:** Paweł Porczyk, Bartłomiej Górski

**Affiliations:** 1 Private Practice, ul. Jedności Narodowej 36, 97-300 Piotrków Trybunalski, Poland; 2 Department of Periodontology and Oral Diseases, Medical University of Warsaw, 02-091 Warsaw, Poland; bartlomiej.gorski@wum.edu.pl

**Keywords:** implant dentistry, bone regeneration, guided bone regeneration, minimally invasive surgery, bone core technique, SPAL, immediate dentoalveolar restoration, esthetic zone, peri-implant defects

## Abstract

**Background/Objectives**: Contemporary implant dentistry is increasingly oriented toward minimally invasive regenerative strategies designed to reduce surgical morbidity while preserving or improving clinical outcomes. Conventional bone augmentation procedures remain effective and biologically well established, but they may be associated with greater patient burden, increased risk of complications, and higher technical demands in selected clinical scenarios. This narrative review critically examines minimally invasive bone regeneration approaches in implant dentistry, with particular focus on the Bone Core Technique, the Sub-Periosteal Peri-implant Augmented Layer (SPAL) technique, and Immediate Dentoalveolar Restoration (IDR), emphasizing their biological rationale, clinical indications, surgical workflows, limitations, and reported outcomes. **Methods**: A structured, non-systematic literature search was conducted in PubMed/MEDLINE, Scopus, and Web of Science to identify publications relevant to minimally invasive bone regeneration in implant dentistry. Priority was given to clinical studies, prospective cohorts, case series, technical descriptions, and biologically oriented conceptual papers addressing vascular preservation, flap limitation, donor-site morbidity, and peri-implant hard- and soft-tissue integration. **Results**: Available evidence suggests that minimally invasive regenerative protocols may offer favorable clinical and patient-centered outcomes only in carefully selected indications and when performed by experienced operators. The strength of support is uneven across techniques: the Bone Core Technique currently has the strongest dedicated prospective follow-up for localized peri-implant defects, SPAL is supported by limited retrospective and emerging histologic evidence, and IDR remains largely based on case reports, technique-driven descriptions, and broader immediate implant literature. **Conclusions**: Minimally invasive bone regeneration reflects a shift toward biologically guided and patient-centered treatment concepts in implant dentistry, but it should not be interpreted as a universal substitute for conventional augmentation. Its successful application depends on careful case selection, sound knowledge of wound healing and defect morphology, and advanced surgical and prosthetic expertise. Further research should prioritize standardized outcome measures, longer follow-up, and comparative prospective studies.

## 1. Introduction

Bone regeneration remains one of the central components of contemporary implant dentistry, particularly in clinical situations characterized by alveolar ridge deficiency, post-extraction remodeling, peri-implant dehiscence-type defects, compromised extraction sockets, or esthetically demanding implant sites [1,2,3]. Over recent decades, guided bone regeneration, staged horizontal and vertical augmentation, and autogenous block grafting have provided highly predictable reconstructive solutions and remain indispensable in advanced defect management [1,2,4]. However, these conventional approaches are often associated with broader flap elevation, increased patient morbidity, multiple surgical stages, longer treatment times, and greater technical burden.

In parallel with the broader movement toward less invasive surgical care, implant dentistry has progressively embraced regenerative concepts intended to reduce surgical trauma while preserving biologic and esthetic outcomes [1,5,6]. However, minimal invasiveness in implant-related bone regeneration should not be interpreted only in procedural terms, such as smaller flap size, flapless access, or avoidance of a secondary donor site. Such a narrow interpretation may be clinically misleading if reduced surgical access compromises the requirements necessary for predictable regeneration.

From a biologic perspective, the value of a less invasive approach depends on whether it preserves the healing factor that is most relevant to a specific defect morphology. Depending on the clinical scenario, this may involve local osteogenic potential, periosteal vascularity, socket architecture, soft-tissue contour support, or prosthetically driven emergence stability [1,5,6,7,8,9]. This indication-specific perspective is particularly relevant in contemporary implant dentistry, where regenerative success is increasingly judged not only by implant survival, but also by contour stability, peri-implant phenotype, patient morbidity, and esthetic integration.

Within this framework, several minimally invasive regenerative approaches have emerged as clinically relevant but distinct strategies. The Bone Core Technique uses local autogenous bone harvested from the implant osteotomy, SPAL emphasizes preservation of the periosteal vascular compartment for buccal contour enhancement, and IDR integrates immediate implant placement with simultaneous reconstruction of compromised esthetic-zone sockets [5,6,10,11]. Although all three are often grouped under the umbrella of minimally invasive regeneration, they differ substantially in rationale, indication range, defect phenotype, evidence level, and dependence on prosthetically driven planning.

Despite growing clinical enthusiasm, the available evidence remains heterogeneous, technique-driven, and unevenly distributed across these approaches [5,6,10,11,12,13,14,15,16,17,18,19]. In addition, conventional guided bone regeneration and staged reconstructive procedures remain biologically driven, clinically validated, and indispensable in advanced defects [1,2,4]. A balanced appraisal is therefore needed to distinguish when limited-access regeneration is appropriate and when reduced invasiveness may risk insufficient reconstruction.

Therefore, the aim of this narrative review is to summarize current minimally invasive bone regeneration approaches in implant dentistry and to organize them within an indication-driven framework integrating defect morphology, vascular preservation, local osteogenic support, immediate socket reconstruction, and prosthetically relevant contour demands. Particular emphasis is placed on the Bone Core Technique, SPAL, and IDR as three representative strategies, with the goal of translating current evidence into clinically meaningful but appropriately cautious decision-making pathways.

## 2. Materials and Methods/Literature Search Strategy

This narrative review was based on a structured, non-systematic literature search conducted in PubMed/MEDLINE, Scopus, and Web of Science to identify publications relevant to minimally invasive bone regeneration in implant dentistry. The search focused primarily on articles published in English from January 2000 to April 2026, while earlier landmark papers were also retained when considered essential for historical or biologic context. The final search update was performed on 5 April 2026. Search terms included combinations of “implant dentistry”, “bone regeneration”, “guided bone regeneration”, “minimally invasive”, “flapless”, “periosteal preservation”, “Bone Core Technique”, “SPAL”, “sub-periosteal peri-implant augmented layer”, “Immediate Dentoalveolar Restoration”, and “IDR”. Reference lists of key publications and recent reviews were manually screened to identify additional relevant studies.

Priority was given to clinical studies, prospective cohorts, retrospective analyses, case series, case reports, and concept-driven articles that addressed biological rationale, technical execution, clinical indications, healing dynamics, and reported outcomes. Studies were prioritized when they provided clear descriptions of defect morphology, surgical workflow, follow-up duration, clinical or radiographic endpoints, esthetic or patient-reported outcomes, and direct relevance to decision-making in implant-related regeneration. Foundational technique papers were retained when they defined the protocol or biological concept, whereas papers focused exclusively on unrelated regenerative techniques, non-implant contexts, or insufficiently described protocols were not prioritized.

Given the narrative and non-systematic design of this review, formal systematic-review methodology, risk-of-bias assessment, certainty-of-evidence grading, and meta-analytic synthesis were not performed. Consequently, the conclusions should be interpreted as a structured critical appraisal rather than as evidence-based clinical recommendations. This methodological choice allows integration of technique descriptions, biologic concepts, and emerging clinical reports, but it also limits the ability to quantify treatment effects, compare interventions directly, or assign definitive levels of evidence across heterogeneous study designs [1,2,5,6,10,11,12,13,14,15,16,17,18,19,20,21,22,23,24,25,26,27,28,29,30,31,32].

## 3. Biological Basis of Minimally Invasive Bone Regeneration

### 3.1. Vascular Supply and Periosteal Preservation

The biological rationale for minimally invasive bone regeneration in implant dentistry is closely linked to preservation of local vascular supply, particularly periosteal vascularization, which contributes to cortical bone nourishment and early wound healing [1,4,6]. Surgical approaches that limit flap elevation and avoid extensive periosteal release may help reduce vascular disruption and maintain favorable local healing conditions, especially at the buccal aspect where thin bone walls and high esthetic demands make vascular preservation clinically relevant [1,3,6,7,8,9,33].

Periosteal preservation may also contribute to wound stability, reduced postoperative morbidity, and integration of grafting materials and peri-implant soft tissues [1,6,8]. Nevertheless, these potential benefits should be interpreted in relation to defect morphology and available evidence; tissue preservation is advantageous only when it does not compromise access, graft stability, or the volume required for predictable regeneration [1,6].

### 3.2. Bone Healing Dynamics in Implant-Related Defects

Bone healing around dental implants is influenced by defect morphology, residual bony walls, implant position, clot stability, graft characteristics, and soft-tissue closure [1,2,3,18,23]. This multifactorial healing environment explains why minimally invasive regenerative approaches are not universally applicable. Their rationale is strongest in localized or contained defects in which local biologic potential can be used without the need for extensive staged reconstruction [1,5,6].

In complex or extensive defects, the limitations of reduced access and restricted graft volume may outweigh the benefits of lower invasiveness [1,2,5]. Therefore, minimally invasive protocols should be understood as indication-sensitive options rather than universal replacements for conventional augmentation procedures [1,2,5].

### 3.3. The Role of Soft-Tissue Management in Regenerative Success

Hard- and soft-tissue outcomes in implant dentistry are closely interrelated, particularly in the esthetic zone, where even small changes in contour or mucosal stability may affect the final clinical result [7,8,9,18,19,23]. For this reason, successful regenerative therapy cannot be reduced to bone gain alone. Even when the primary goal is hard-tissue reconstruction, peri-implant soft-tissue thickness, contour stability, wound closure, and long-term mucosal integration strongly influence both biologic and esthetic outcomes [7,8,9].

Minimally invasive protocols may reduce soft-tissue trauma and support esthetic integration in selected cases, particularly when regeneration is performed simultaneously with implant placement [6,8,9,30,31]. However, soft-tissue thickness, contour stability, wound closure, and prosthetic support remain critical determinants of outcome, and these factors should be evaluated together with hard-tissue reconstruction rather than considered secondary to bone gain alone [7,8,9,30].

### 3.4. Technique-Specific Soft-Tissue Handling and Long-Term Contour Stability

Soft-tissue handling differs among the reviewed minimally invasive techniques because each protocol relies on a different biologic compartment and a different mechanism of contour stabilization. In the Bone Core Technique, the primary soft-tissue objective is to obtain sufficient access for atraumatic retrieval, adaptation, and fixation of the autogenous core while avoiding unnecessary extension of the flap. Papillae, marginal mucosa, and adjacent periosteal attachments should be preserved whenever possible; sharp retraction, overheating during osteotomy preparation, excessive flap stretching, and uncontrolled contact between rotary instruments and the soft-tissue envelope should be avoided. In this technique, long-term soft-tissue contour stability is expected to depend mainly on preservation of the peri-implant mucosal envelope, stable bone-core adaptation, limited scar formation, and subsequent maturation of keratinized tissue when required [1,5,7,8,9].

In SPAL, soft-tissue management is central to the biologic concept. The subperiosteal pouch should be prepared with sufficient thickness to preserve the periosteal vascular layer, but without excessive thinning, tearing, or perforation of the periosteal flap. Tension control is critical: the volume of particulate or block xenograft should not exceed the elastic capacity of the subperiosteal compartment, because overfilling may increase the risk of periosteal perforation, flap tension, mucosal ischemia, wound dehiscence, or contour instability. When adequate passive adaptation of the periosteal layer cannot be achieved, reducing graft volume or selecting a staged reconstructive approach may be biologically safer. In the long term, SPAL-related contour stability is likely influenced by the quality of periosteal preservation, graft immobilization, mucosal thickness, and the restorative contour after loading [6,7,8,9,12,13,14,20,21,22].

In IDR, the soft-tissue strategy is inseparable from immediate implant positioning, graft placement, and provisionalization. Depending on the clinical protocol and socket morphology, complete primary closure is not always the main objective; instead, a stable biologic and prosthetic seal may be achieved through atraumatic extraction, preservation of papillae, tuberosity-derived hard- and/or soft-tissue grafting, graft containment, and a carefully shaped provisional restoration that supports but does not compress the peri-implant mucosa. Open or semi-open healing may be acceptable only if the grafted socket is stable, protected from collapse, and isolated from excessive mechanical pressure. Over-contoured provisional restorations, inadequate emergence-profile design, residual infection, or unstable buccal reconstruction may increase the risk of recession, buccal contour loss, and esthetic failure [7,8,9,10,11,15,16,17,30,31].

Across techniques, the long-term stability of the final soft-tissue contour should be interpreted as the combined result of surgical tissue preservation, graft stability, mucosal thickness, keratinized-tissue quality, emergence-profile design, and loading-related restorative support. Therefore, future studies should not report hard-tissue gain alone, but should also include standardized soft-tissue thickness, mucosal recession, papilla fill, keratinized mucosa, buccal contour, digital volumetric change, and patient-reported esthetic outcomes over time [7,8,9,30,31,34].

## 4. Rationale for Minimally Invasive Regenerative Approaches in Implant Dentistry

### 4.1. Limitations of Conventional Augmentation Techniques

Conventional regenerative techniques remain essential in implant dentistry, particularly in the management of severe horizontal or vertical ridge deficiencies [1,2,4]. Guided bone regeneration, block grafting, and staged reconstructive approaches can provide substantial clinical benefit and remain highly effective when properly indicated [1,2]. However, these methods often require broader flap elevation, additional donor sites, larger biomaterial volumes, multiple surgical stages, and extended healing times [1,2]. Such features may increase treatment burden, postoperative discomfort, and the overall technical sensitivity of the procedure [1,2,4].

The growing interest in minimally invasive regenerative concepts did not emerge because conventional augmentation became obsolete, but because clinicians increasingly recognized that, in selected cases, therapeutic goals might be achieved with reduced tissue trauma and lower morbidity [1,2]. Accordingly, the rationale for minimally invasive bone regeneration is not based on rejecting conventional surgery, but on refining the balance between reconstructive control, biologic preservation, and patient burden [1].

### 4.2. Patient-Centered Benefits: Morbidity, Recovery, and Surgical Burden

One potential attraction of minimally invasive regenerative strategies is the possibility of reducing postoperative discomfort, shortening recovery time, limiting donor-site morbidity, and decreasing surgical burden for the patient [5,6,10,30,34]. These considerations are relevant in implant dentistry, especially in elective and esthetically sensitive treatments [18,19,30,34]. However, direct comparative evidence for patient-centered benefits remains limited and should be interpreted cautiously.

Minimally invasive regenerative protocols may align with contemporary patient-centered care when they reduce burden without compromising essential regenerative goals. This depends heavily on strict case selection, adequate defect morphology, and appropriate surgical expertise [5,6,10,30].

### 4.3. Defect Morphology and Case Selection Principles

Case selection remains the defining factor in the use of minimally invasive regenerative approaches [1,5,6,10]. Localized peri-implant defects, contained buccal dehiscence-type deficiencies, and highly selected extraction sockets in the esthetic zone differ fundamentally from extensive ridge deficiencies requiring staged augmentation and larger-volume reconstruction [1,5,6,10,18,19]. In the former situations, tissue-preserving strategies may be appropriate; in the latter, conventional reconstructive approaches may provide greater control and predictability [1,2].

**Figure 1:**
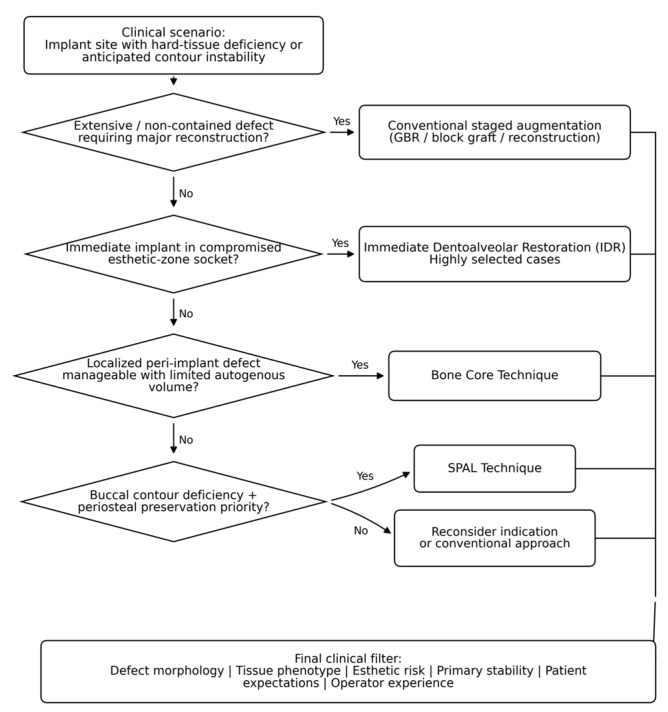
Indication-driven clinical decision-making pathway for minimally invasive bone regeneration in implant dentistry. The algorithm integrates defect morphology, implant timing, and biologic priorities to guide selection between Bone Core Technique, SPAL, Immediate Dentoalveolar Restoration (IDR), and conventional augmentation approaches. Final treatment decisions should incorporate patient-specific factors and operator experience.

The relevance of defect morphology also explains why currently proposed minimally invasive techniques should not be considered interchangeable. Each addresses a different clinical scenario within a limited indication range. Therefore, clinical success depends less on reduced invasiveness itself and more on whether the defect characteristics permit regeneration under favorable conditions [1,3,5,6]. A simplified decision-making pathway is proposed in Figure 1 as a conceptual clinical aid rather than a formal guideline.

## 5. Bone Core Technique

### 5.1. Biological Concept and Surgical Rationale

The Bone Core Technique is based on the use of an autogenous bone cylinder harvested from the implant osteotomy and repositioned to augment a localized peri-implant defect [5]. Its biologic attractiveness lies in the use of fresh autogenous bone obtained from the same surgical field, thereby preserving osteogenic potential while avoiding the morbidity associated with a second donor site [5]. This makes the technique conceptually appealing in limited defects, where a small amount of biologically active autogenous tissue may be sufficient to support simultaneous implant placement and localized augmentation [5].

Within the framework proposed in this review, the biologic determinant primarily preserved by the Bone Core Technique is localized osteogenic support derived from the implant osteotomy itself.

From a biologic standpoint, the Bone Core Technique reflects a highly conservative use of autogenous tissue. Rather than harvesting bone from a distant intraoral site, it utilizes tissue already available within the implant preparation itself, thereby integrating graft procurement and implant placement into a single procedure [5]. In this sense, it represents one of the clearest examples of minimally invasive regenerative thinking in implant dentistry.

### 5.2. Surgical Protocol and Technical Considerations

The surgical workflow of the Bone Core Technique combines implant bed preparation and graft harvesting within the same operative event [5]. After careful osteotomy preparation, the autogenous bone core is retrieved and adapted to the localized defect site in conjunction with implant placement [5]. Because the volume of harvested tissue is inherently limited, the procedure requires precise defect assessment and meticulous adaptation of the bone core to ensure adequate stability and intimate contact with the recipient area [5].

**Figure 2:**
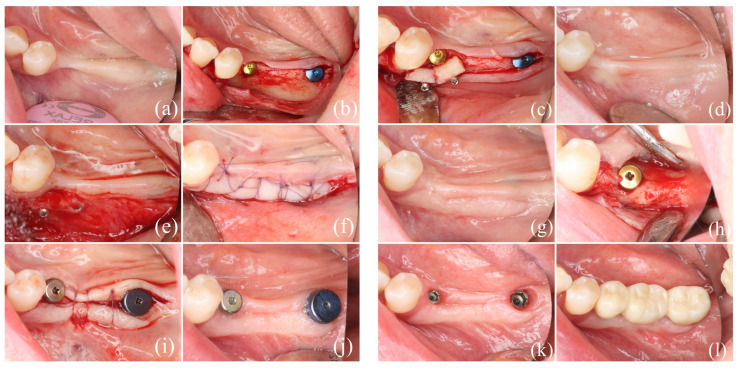
Representative clinical case illustrating the described minimally invasive bone regeneration protocol. (**a**) Preoperative clinical view. (**b**) Intraoperative view following implant placement. (**c**) Fixation of the autogenous bone graft harvested during implant site preparation. (**d**) Postoperative clinical view at 3 months. (**e**) Removal of the graft fixation screws. (**f**) Free gingival graft procedure. (**g**) Clinical view at 4.5 months postoperatively. (**h**) Re-entry of the surgical site. (**i**) Replacement of the cover screws with healing abutments. (**j**) Clinical view at 6 months postoperatively. (**k**) Soft-tissue emergence profile around the implants. (**l**) Final screw-retained implant-supported prosthetic restoration.

**Figure 3:**
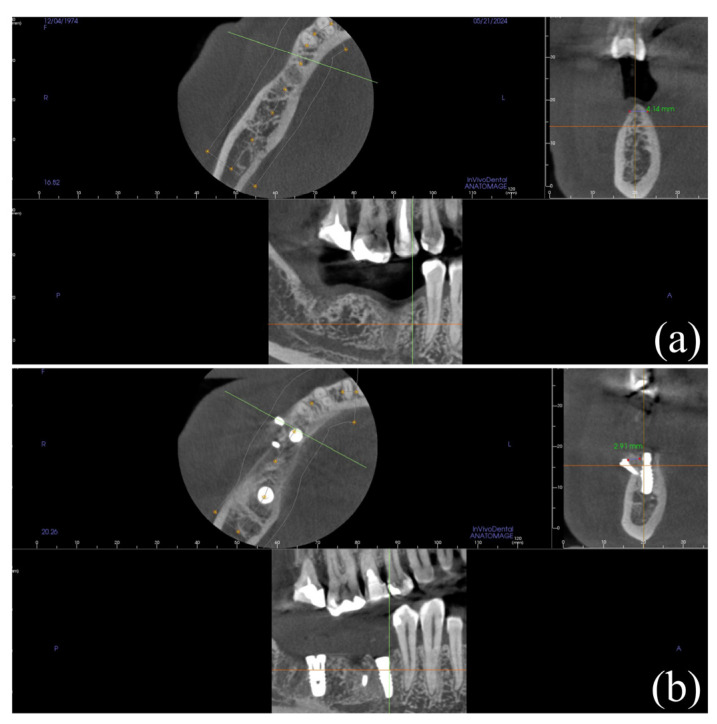
Radiographic evaluation of the clinical case shown in Figure 2. (**a**) Preoperative radiographic view. (**b**) Postoperative radiographic view after implant placement and augmentation. Colored lines are used only as visual reference markers and do not represent additional quantitative measurements.

These technical characteristics also define the boundaries of the method. The procedure is not intended for large-volume reconstruction, but rather for targeted correction of limited defects in which local autogenous augmentation may be sufficient [1,5]. As such, the success of the technique is closely tied to proper case selection, surgical precision, and the ability to obtain adequate regenerative support without overextending the indication. A representative clinical case treated using the described minimally invasive technique is shown in Figure 2, while Figure 3 shows the corresponding radiographic images from the same case.

### 5.3. Indications, Advantages, and Limitations

The Bone Core Technique appears most suitable for localized peri-implant dehiscence-type defects and other situations in which limited autogenous augmentation can support implant placement [5]. Its main advantages include avoidance of a second donor site, preservation of autogenous biological properties, and reduced overall surgical invasiveness [5]. In selected cases, it may therefore provide a targeted alternative to more extensive augmentation procedures, although this interpretation remains indication-dependent [1,5].

At the same time, the limitations of the method are clear. The available graft volume is restricted, its applicability depends heavily on favorable defect morphology, and it is unlikely to provide sufficient reconstructive control in larger horizontal or vertical deficiencies [1,5]. Accordingly, the Bone Core Technique should be regarded as a targeted minimally invasive solution rather than a general-purpose augmentation strategy [1,5].

### 5.4. Available Clinical Evidence

Among the minimally invasive regenerative approaches discussed in this review, the Bone Core Technique has one of the more substantial dedicated clinical datasets [5]. A prospective study included 186 consecutively treated patients and reported at least 5 years of clinical and radiographic follow-up, suggesting favorable long-term outcomes in appropriately selected defects [5]. This gives the technique a relatively stronger evidence base than SPAL and IDR, but the available support remains protocol-specific and indication-restricted [5].

Nevertheless, the available evidence remains technique-centered and should not be interpreted as proof of superiority over conventional regenerative procedures [1,2,5]. Although the reported outcomes are encouraging, additional comparative studies would be needed to determine whether the benefits of reduced invasiveness translate into consistently equivalent or improved clinical results across broader patient populations [1,2].

## 6. Sub-Periosteal Peri-Implant Augmented Layer (SPAL) Technique

### 6.1. Biological Concept and Preservation of Periosteal Blood Supply

In conceptual terms, SPAL is designed to preserve the periosteal vascular envelope as the local factor most relevant to buccal contour healing and tissue stability in selected peri-implant defects.

The SPAL technique was introduced as an approach to correcting peri-implant buccal bone dehiscence while preserving periosteal blood supply [6]. Its biologic premise is that augmentation beneath a preserved periosteal layer may support hard- and soft-tissue thickening while minimizing the vascular compromise associated with more extensive flap elevation and periosteal release [6,12]. In this respect, SPAL is one of the most explicitly periosteal-preserving concepts among contemporary minimally invasive regenerative approaches.

This rationale is particularly relevant in areas where buccal contour stability is clinically important and where excessive flap manipulation may negatively affect hard- or soft-tissue outcomes [6,7,8,9]. However, the potential advantage of preserving the periosteal envelope should be interpreted together with defect size, tissue phenotype, graft stability, and the clinician’s ability to control the subperiosteal compartment [6,12].

### 6.2. Surgical Workflow and Biomaterial Considerations

SPAL generally involves the creation of a subperiosteal pouch or layer in the buccal peri-implant region, placement of graft material, and preservation of tissue integrity with minimal flap manipulation [6,12,20]. Published reports describe the use of xenograft-based materials, including particulate deproteinized bovine bone mineral and bovine-derived block materials, in conjunction with this protocol [14,20,22]. Unlike the Bone Core Technique, which relies on locally harvested autogenous bone, SPAL is primarily focused on optimizing a preserved biologic compartment for augmentation [6,20].

**Figure 4:**
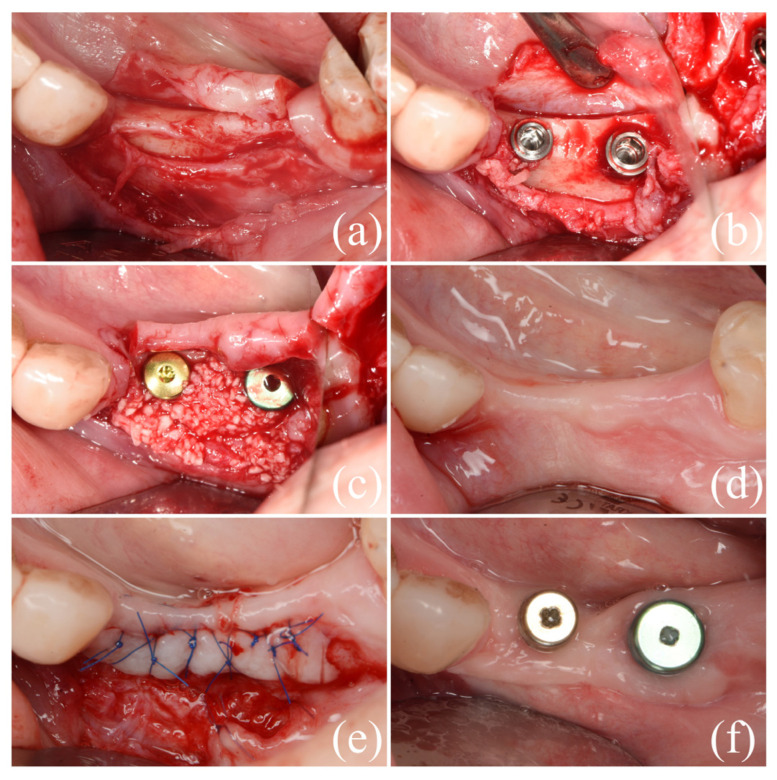
Representative clinical case illustrating the described minimally invasive bone regeneration protocol. (**a**) Preparation of a split-thickness flap and subperiosteal pouch for graft placement. (**b**) Implant placement. (**c**) Placement of the grafting material into the subperiosteal pouch. (**d**) Clinical view 3 months postoperatively. (**e**) Free gingival graft procedure. (**f**) Final outcome following hard- and soft-tissue augmentation.

**Figure 5:**
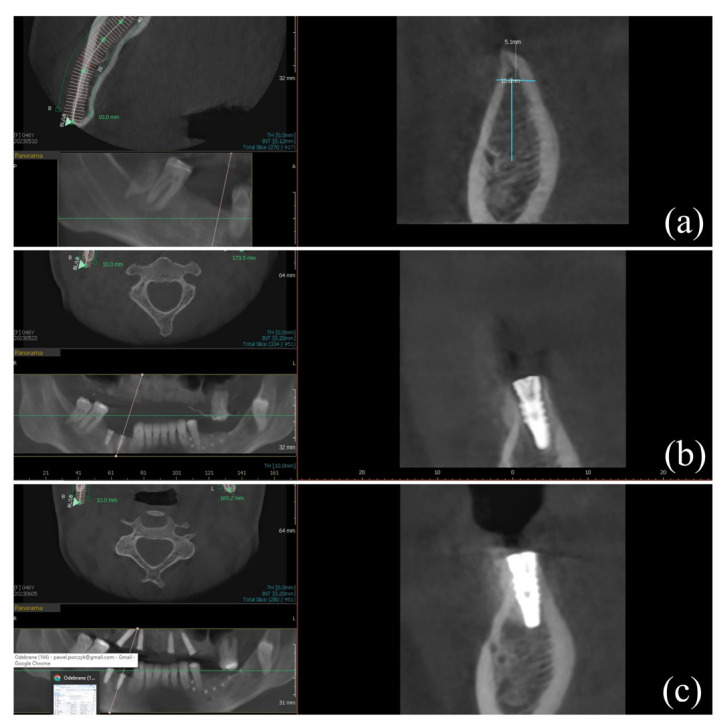
Radiographic evaluation of the clinical case shown in Figure 4, demonstrating the treatment site before and after minimally invasive bone regeneration. (**a**) Before (**b**) after surgery (**c**) 3 months after surgery. Colored lines are used only as visual reference markers and do not represent additional quantitative measurements.

This distinction is important because the success of SPAL depends not only on the graft material itself but also on delicate handling of the periosteal plane and maintenance of the subperiosteal compartment [6,12]. The technique is therefore biologically elegant but surgically sensitive, and its predictability is likely influenced by both local anatomy and operator experience. A representative clinical case treated using the described minimally invasive technique is shown in Figure 4, while Figure 5 shows the corresponding radiographic images from the same case.

### 6.3. Indications, Contraindications, and Technique Sensitivity

SPAL appears particularly relevant for correction of peri-implant buccal dehiscence and enhancement of buccal contour where periosteal preservation is desirable [6,13,21]. This may make it useful when hard- and soft-tissue thickening is required but extensive flap release is considered undesirable [6,13]. However, the technique should be regarded as highly indication-sensitive and operator-dependent rather than broadly reproducible without specific training [6,12,13,21].

Thin tissue phenotypes, complex defect morphologies, and insufficient control of the periosteal plane may compromise the predictability of the procedure [6,7,8,9]. As with other minimally invasive approaches, reduced access does not necessarily mean reduced complexity. On the contrary, limited exposure may increase the technical demands of the surgery and reduce the margin for error [6,12].

### 6.4. Available Clinical and Histologic Evidence

The current evidence base for SPAL is still limited but expanding [6,12,13,14,20,21,22]. The literature includes the original technical description, refinements in soft-tissue management, retrospective clinical observations, application in peri-implantitis lesions, and a recent histologic case report [6,12,13,14,20,21,22]. Together, these publications support the biologic plausibility of SPAL and suggest potential buccal augmentation benefits in selected peri-implant defects, but they do not yet establish predictable effectiveness across broader clinical settings [6,12,13,14,20,21,22].

However, comparative long-term clinical data remain scarce [1,6,12,13,14,20,21,22]. Most available studies are case-based, retrospective, or technique-driven, which limits the strength of conclusions that can be drawn regarding generalizability and reproducibility [6,12,13,14,20,21,22]. Thus, while SPAL is one of the more intriguing biologically oriented techniques in the field, it still requires stronger validation before it can be considered a standardized therapeutic option [1].

## 7. Immediate Dentoalveolar Restoration (IDR)

From a framework perspective, IDR primarily targets immediate preservation or reconstruction of dentoalveolar architecture in high-risk esthetic settings.

### 7.1. Biological and Esthetic Rationale

IDR is primarily used in extraction sockets in the esthetic zone, where immediate implant placement is combined with simultaneous reconstruction of deficient dentoalveolar structures [10,11,18,19]. Its rationale is to preserve or restore buccal architecture at an early stage, prevent ridge-contour collapse, and integrate regenerative therapy with immediate implant treatment when strict anatomic, prosthetic, and surgical conditions are fulfilled [3,10,11,18,23].

**Figure 6:**
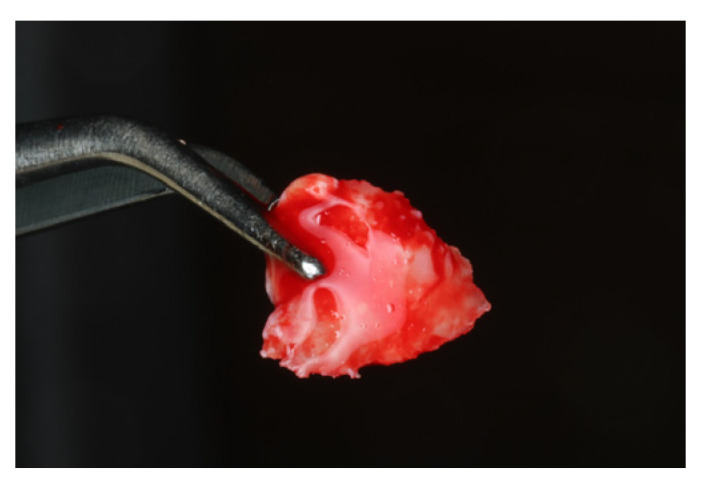
Autogenous bone graft harvested from the maxillary tuberosity.

This concept is relevant in esthetic implant dentistry because treatment success depends not only on implant integration but also on buccal contour stability, mucosal harmony, and emergence-profile control [7,8,9,18,19]. In this context, IDR can be viewed as an expert-driven attempt to address biologic deficiency and esthetic risk during the same surgical event [10,11]. An autogenous bone graft harvested from the maxillary tuberosity is shown in Figure 6, representing a key biologic component of the IDR protocol.

### 7.2. Immediate Implant Placement and Simultaneous Reconstruction

The clinical workflow of IDR typically combines extraction, implant placement, management of the buccal defect or gap, and simultaneous reconstruction of hard and soft tissues in a time-sensitive esthetic setting [10,11,15,16,17]. This integrated strategy may reduce total treatment time and may help maintain native architecture in selected situations [10,11,18,19]. However, the attractiveness of a one-stage approach should be balanced against its high technical complexity and limited protocol-specific evidence.

**Figure 7:**
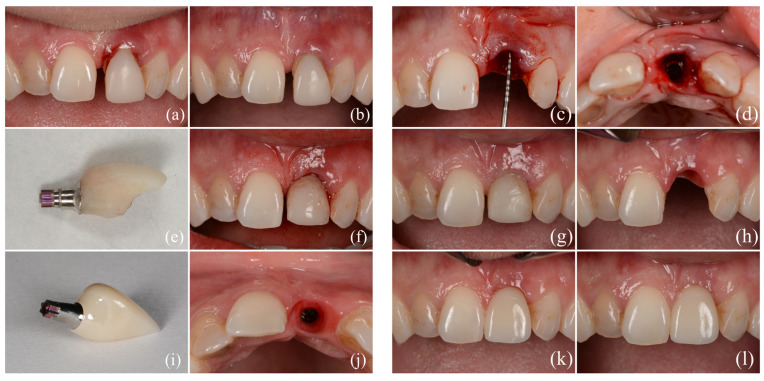
Representative clinical case illustrating the described minimally invasive bone regeneration protocol. (**a**) Preoperative clinical situation of tooth 21 with a vertical root fracture. (**b**) Preoperative antibiotic therapy with amoxicillin/clavulanic acid 875/125 mg twice daily, started 3 days before surgery. (**c**) Measurement of the buccal plate defect with a periodontal probe after tooth extraction. (**d**) Implant placed in a prosthetically driven position, maintaining adequate space for an autogenous bone graft harvested from the maxillary tuberosity. (**e**) Provisional restoration fabricated using a temporary abutment and composite resin. (**f**) Clinical situation after placement of the autogenous bone graft harvested from the maxillary tuberosity and delivery of the provisional restoration. (**g**) Clinical situation 2 weeks after surgery. (**h**) Emergence profile 3 months after surgery. (**i**) Final zirconia restoration fabricated on a standard titanium base. (**j**) Emergence profile and soft-tissue contour 3 months after surgery. (**k**) Delivery of the final restoration. (**l**) Clinical situation 1 year after delivery of the final screw-retained restoration.

**Figure 8:**
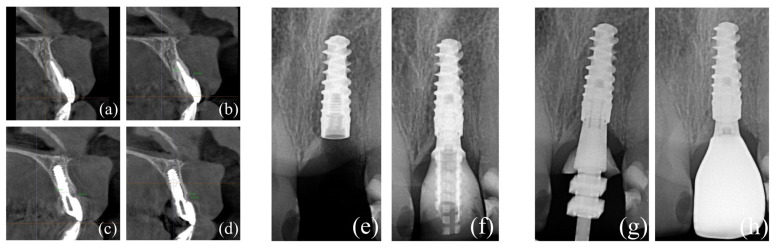
Corresponding radiographic images of the clinical case presented in Figure 7. (**a**) Pre-fracture CBCT image. (**b**) CBCT image following vertical root fracture. (**c**) CBCT image after implant placement, fixation of the autogenous bone graft harvested from the maxillary tuberosity, and placement of the provisional restoration. (**d**) CBCT image obtained 3 months postoperatively. (**e**) Intraoral radiograph after implant placement. (**f**) Intraoral radiograph after placement of the provisional restoration. (**g**) Intraoral radiograph 3 months postoperatively. (**h**) Intraoral radiograph after delivery of the final restoration. Colored lines are used only as visual reference markers and do not represent additional quantitative measurements.

At the same time, IDR condenses several biologically and technically critical steps into a single surgical event [10,11,15,16,17]. This includes atraumatic extraction, three-dimensional implant positioning, primary stability, reconstruction of the compromised socket, provisionalization, and control of the buccal contour. As a result, the procedure is highly technique-sensitive and less forgiving than staged approaches in cases with unfavorable anatomy, inadequate primary stability, or limited prosthetic control [10,11,15,16,17]. A representative clinical case treated using the described minimally invasive technique is shown in Figure 7, while Figure 8 shows the corresponding radiographic images from the same case.

### 7.3. Soft-Tissue Integration and Esthetic Stability

The success of IDR depends heavily on soft-tissue integration and esthetic stability [7,8,9,10,11,30,31]. Even when osseointegration is achieved, inadequate management of buccal contour, mucosal thickness, or emergence profile may compromise the final esthetic result [7,8,9,23,26,30]. For this reason, soft-tissue considerations are not secondary in IDR but are central to the rationale and execution of the technique [10,11].

This emphasis on esthetic stability also contributes to the operator dependence of IDR. Favorable outcomes reported in expert hands may reflect not only the biologic validity of the concept but also a high level of surgical and prosthetic control [10,11,30]. Therefore, published IDR outcomes should be interpreted cautiously and should not be assumed to be reproducible with equal predictability in routine clinical settings.

### 7.4. Clinical Indications, Limitations, and Outcomes

IDR is most applicable in highly selected esthetic-zone cases in which immediate implant placement is feasible, primary stability can be achieved, implant positioning can be prosthetically controlled, and simultaneous reconstruction may help maintain or restore ridge contour [10,11,18,19]. Its main advantages include reduced treatment time and early management of dentoalveolar architecture [10,11,18,19]. These features make it appealing in compromised sockets where esthetic risk is substantial, but only when strict indication criteria are met [10,11].

Its limitations, however, are equally important. IDR is characterized by pronounced technique sensitivity, dependence on primary stability, demanding provisionalization, and difficulty in standardizing protocols across operators and defect morphologies [10,11,15,16,17,18,19,34]. Moreover, the available IDR-specific literature remains dominated by case reports, technique-driven descriptions, and expert-based clinical narratives [10,11,15,16,17]. Although broader evidence on immediate implant placement supports feasibility in selected esthetic-zone cases, this should not be conflated with direct proof of IDR-specific superiority [18,19].

Importantly, a substantial portion of the evidence frequently cited in support of IDR actually derives from the broader immediate implant literature rather than from protocol-specific IDR validation. This distinction is essential when interpreting reported esthetic and clinical outcomes [18,19,23,24,25,26,27,28,29].

## 8. Comparative Appraisal of Minimally Invasive Techniques

### 8.1. Invasiveness Versus Regenerative Potential

The central message emerging from this review is that minimal invasiveness in implant-related bone regeneration should not be defined solely by flap size, surgical access, or the absence of a secondary donor site. Its clinical value depends on whether reduced invasiveness is compatible with adequate regenerative control in a given defect morphology [1,5,6,10,11,12].

Accordingly, comparison of the Bone Core Technique, SPAL, and IDR is most useful when framed as a balance between reduced invasiveness and achievable regenerative control. Bone Core primarily provides localized autogenous osteogenic support, SPAL prioritizes periosteal vascular preservation and buccal contour support, and IDR targets immediate maintenance or reconstruction of socket architecture in high-risk esthetic settings [5,6,10,11,12].

### 8.2. Morbidity, Esthetic Outcomes, and Patient-Reported Benefits

All three techniques aim, directly or indirectly, to reduce surgical trauma and improve patient-centered outcomes [5,6,10,30,34]. Potential benefits include less postoperative discomfort, reduced donor-site morbidity, shorter recovery, and greater treatment acceptance, particularly in esthetic or elective implant scenarios [5,6,10,30]. Nevertheless, the current literature provides limited direct comparative support for these patient-centered advantages.

However, direct comparative data on pain, swelling, analgesic consumption, treatment burden, return to work, esthetic satisfaction, willingness to repeat treatment, and recovery remain sparse [19,30,31,34]. Esthetic outcomes are also reported inconsistently, and validated measures of contour stability, patient-reported satisfaction, and volumetric change are not used uniformly across studies [8,9,19,30,31,34].

### 8.3. Technique Sensitivity and Learning Curve

A recurring issue across the literature is that minimally invasive procedures are not necessarily simple procedures [5,6,10,11,12,33]. Bone core harvesting requires precise adaptation and indication control, SPAL requires delicate management of the periosteal compartment, and IDR demands high-level coordination of extraction, immediate implant positioning, reconstruction, provisionalization, and esthetic contour control [5,6,10,11,12].

These technical demands limit direct extrapolation of favorable outcomes from expert centers to routine practice [5,6,10,11,34]. The learning curve should therefore be considered part of the clinical appraisal of each technique rather than a secondary practical concern.

Operator dependence is particularly relevant for outcome interpretation and training. Limited surgical access may reduce visual control, increase dependence on tactile feedback and preoperative planning, and narrow the margin for intraoperative correction. For this reason, future studies should report operator experience, training background, case volume, and protocol standardization, because these variables may influence both reproducibility and complication rates [5,6,10,11,12,33,34].

#### Technique-Specific Technical Thresholds, Learning Curve, and Complications

A clinically realistic appraisal of minimally invasive regeneration should include the threshold for safe application, not only the biologic rationale. For the Bone Core Technique, the operator should be experienced in implant osteotomy design, atraumatic retrieval of an autogenous bone cylinder, three-dimensional implant positioning, and rigid fixation of small autogenous grafts. Required armamentarium may include core drills or trephines, fixation screws or pins, microsurgical retractors, magnification, and careful irrigation. Specific complications include fracture or fragmentation of the bone core, insufficient graft volume, overheating, poor adaptation to the recipient defect, fixation instability, flap trauma during core retrieval, and residual dehiscence if the indication is overextended [1,5].

For SPAL, the technical threshold is primarily related to precise preparation of the subperiosteal compartment. Operators should be familiar with split-thickness flap management, periosteal plane identification, tunnel or pouch instrumentation, and biomaterial stabilization under limited visual access. Magnification, microsurgical blades, fine elevators, and delicate periosteal instruments may improve control. Specific complications include periosteal perforation, pouch collapse, graft migration, excessive flap tension, wound dehiscence, mucosal thinning, infection, and insufficient buccal contour gain when the defect is too large or the periosteal compartment cannot be maintained [6,12,13,14,20,21,22].

For IDR, the clinical threshold is particularly high because extraction, immediate implant placement, buccal reconstruction, soft-tissue management, and provisionalization are combined in one surgical event. Operator experience should include atraumatic extraction, immediate implant placement in the esthetic zone, maxillary tuberosity graft harvesting, socket reconstruction, screw-retained provisionalization, and prosthetically driven emergence-profile design. Specific complications include buccal wall collapse, loss of primary stability, graft displacement, mucosal recession, papilla loss, provisional restoration overpressure, infection, and failure to maintain the planned three-dimensional contour [10,11,15,16,17,18,19,23,24,25,26,27,28,29,30,31].

The available literature does not define a validated number of procedures required to complete the learning curve for any of these techniques. Therefore, any numerical threshold would be speculative. Until learning curves are quantified, clinical studies should report operator training, previous case volume, use of magnification or digital planning, protocol standardization, and intraoperative complications. From a training perspective, these techniques should be adopted after competence has been achieved in conventional GBR, implant positioning, soft-tissue surgery, and management of complications, rather than being treated as simplified alternatives for inexperienced operators [5,6,10,11,12,13,14,15,16,17,20,21,22,33,34]. Technique-specific clinical thresholds, learning curve considerations and potential complications are summarised in Table 1.

Because published studies do not provide validated numerical learning-curve thresholds, this table is intended as an interpretive clinical appraisal rather than a formal competency guideline.

### 8.4. Prosthetically Driven Risk and Contour Support

Across all three minimally invasive approaches, long-term success is not determined by bone gain alone, but by the interaction between regenerated tissues, implant three-dimensional positioning, abutment transition, emergence profile, and prosthetic support of contour stability [7,8,9,30,31,34]. This relationship is particularly relevant for SPAL and IDR, where buccal contour maintenance is inseparable from restorative design and soft-tissue support.

From a clinical perspective, prosthetically driven risk becomes especially important when emergence-profile pressure, transition-zone design, crown contour, provisional restoration geometry, or inadequate soft-tissue support may compromise otherwise acceptable regenerative outcomes. In SPAL, restorative contour may influence whether the augmented buccal envelope is maintained after loading; in IDR, provisionalization and final emergence profile are central to preservation of the reconstructed socket contour. Future studies should therefore integrate restorative variables and digital contour superimposition into both outcome reporting and interpretation.

### 8.5. Current Gaps in Evidence

Across all three approaches, major evidence gaps remain [5,6,10,11,12,13,14,15,16,17,18,19,20,21,22]. Study designs are heterogeneous, sample sizes are often limited, follow-up periods vary, and standardized outcomes such as defect morphology, buccal bone thickness, volumetric contour stability, esthetic indices, complication rates, and patient-reported morbidity are inconsistently reported [5,6,8,9,10,11,12,13,14,15,16,17,19,20,21,22,30,31,34].

At present, the Bone Core Technique has the strongest dedicated prospective follow-up, SPAL has an expanding but still limited evidence base, and IDR remains largely supported by case-based and technique-oriented literature [5,6,10,11,12,13,14,15,16,17,20,21,22]. Accordingly, all three approaches may be clinically useful in selected indications, but none should be interpreted as a universally validated standard of care. The key characteristics of the three discussed techniques are summarized in Table 2, while Table 3 provides a structured synthesis of the available evidence, including study type, follow-up, main outcomes reported, and key limitations. For clarity, the relative level of support, clinical promise, and unresolved limitations of the three techniques are summarized in Table 4.

The evidence base remains heterogeneous and unevenly distributed across techniques. Bone Core currently has the strongest dedicated prospective follow-up, SPAL is supported by limited retrospective and emerging histologic data, and IDR remains predominantly case-based and highly operator-dependent.

Interpretive evidence grading in this table is intended to support critical clinical appraisal rather than provide a formal evidence-based guideline classification.

### 8.6. Conceptual Classification and Indication-Driven Clinical Translation

**Figure 9:**
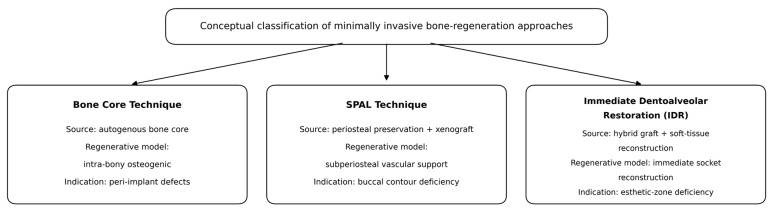
Conceptual Classification of Minimally Invasive Bone Regeneration Approaches in Implant Dentistry.

To move beyond a purely descriptive use of the term minimally invasive, Figure 9 proposes a conceptual classification of the reviewed techniques across three complementary axes: source-driven logic, biology-driven preservation, and indication-driven application. In this framework, Bone Core can be understood as a source-driven local autogenous strategy, SPAL as a compartment-based periosteal-preserving strategy, and IDR as a hybrid immediate reconstruction strategy tailored to compromised esthetic sockets.

The framework is intended as an interpretive aid rather than a formal evidence-based classification. It emphasizes that the reviewed techniques are not interchangeable low-morbidity alternatives, but distinct strategies with different biologic targets and different levels of supporting evidence.

To facilitate indication-sensitive clinical translation, Table 5 summarizes a proposed decision matrix integrating defect morphology, biologic priorities, esthetic demands, procedural complexity, and the continuing role of conventional regenerative approaches. This matrix should be interpreted cautiously because it is derived from heterogeneous evidence and expert-level synthesis rather than from direct comparative trials.

This matrix is proposed as an indication-sensitive clinical aid and should not be interpreted as a formal treatment guideline or an evidence-based algorithm.

### 8.7. When Minimal Invasiveness Becomes Biological Under-Treatment

A major practical implication of the proposed framework is that reduced surgical access can become biologic under-treatment when the defect exceeds the regenerative capacity of a limited intervention. Scenarios in which conventional staged augmentation may remain biologically superior include extensive three-dimensional ridge collapse, vertical defects greater than approximately 4–5 mm, combined horizontal and vertical deficiency, absence of palatal or lingual bony support, severe soft-tissue deficiency, inability to achieve ideal three-dimensional implant position or primary stability, and thin phenotypes with high esthetic risk or insufficient soft-tissue planning [1,2,7,8,9].

### 8.8. Operationalizing Biological Treatment Inadequacy for Clinical Decision-Making

For clinical use, biological treatment inadequacy can be operationalized as a mismatch between the reconstructive demand of the defect and the regenerative capacity of a limited-access procedure. This mismatch should be assessed before selecting a minimally invasive technique. The most relevant variables are defect morphology, defect extent, integrity of the remaining bony walls, soft-tissue phenotype, implant-position feasibility, and the ability to stabilize the graft and soft-tissue envelope.

**Figure 10:**
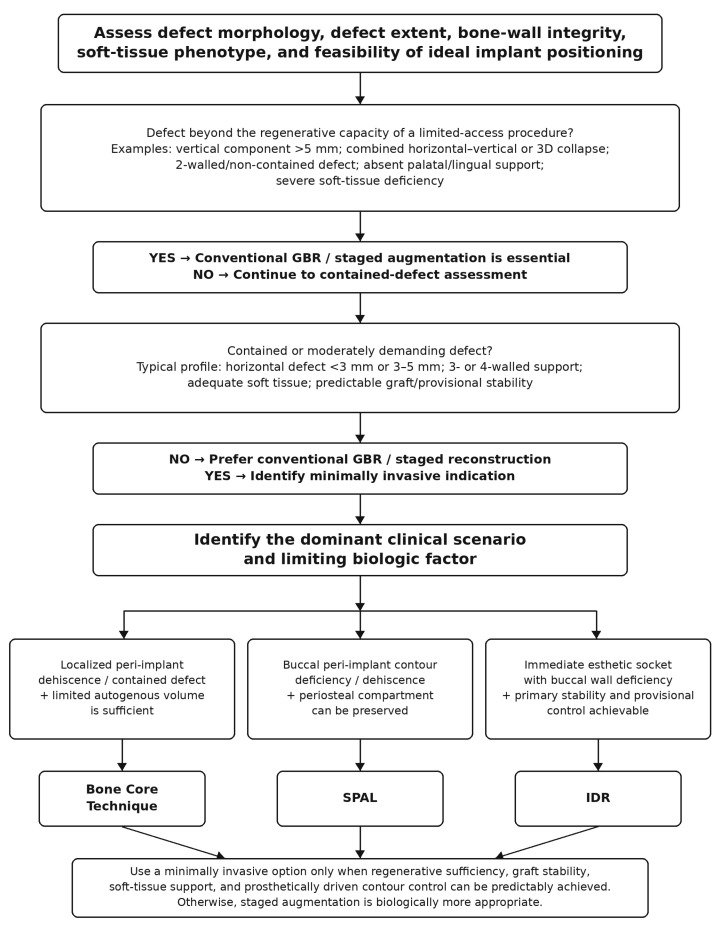
Operational decision flowchart for avoiding biological treatment inadequacy in minimally invasive bone regeneration.

Within this framework, minimally invasive techniques may be clinically preferable in contained or localized defects where the limiting biologic factor can be addressed without extensive flap elevation or large-volume reconstruction. This should not be interpreted as proven superiority over conventional GBR; rather, it indicates situations in which a limited approach may offer an advantageous balance between regenerative sufficiency and reduced morbidity. Conversely, traditional staged augmentation should be considered essential when defect morphology requires spatial control, vertical support, soft-tissue expansion, or graft stability that cannot be predictably achieved through a limited-access approach [1,2,7,8,9]. The operational decision flowchart is presented in Figure 10.

This flowchart is proposed as a practical decision aid. It should be applied together with patient-specific risk factors, esthetic demands, infection control, restorative planning, and operator experience, and it should not be interpreted as a formal evidence-based guideline.

## 9. Discussion

Minimally invasive bone regeneration in implant dentistry reflects a broader shift toward biologically guided and patient-centered therapeutic concepts. However, the term minimally invasive should be interpreted cautiously. Reduced flap size, limited access, or avoidance of a secondary donor site are clinically meaningful only when they preserve adequate regenerative control for a specific defect morphology [1,5,6,10,11,12].

The conceptual framework proposed in Figure 9 and the indication-driven matrix presented in Table 5 support a structured but non-definitive interpretation of these approaches. Bone Core, SPAL, and IDR represent different strategies: localized autogenous osteogenic support, periosteal vascular preservation, and immediate dentoalveolar architecture preservation. Their common denominator is not reduced access alone, but selective application in defects where the chosen technique can reasonably meet the biologic and prosthetic demands of treatment.

The available literature suggests that these approaches are most valuable when used in carefully selected indications rather than as substitutes for conventional augmentation. Bone Core has the strongest dedicated clinical support among the reviewed techniques and appears best suited for localized peri-implant defects where limited autogenous volume is sufficient [1,5]. This advantage should not be extrapolated to larger horizontal or vertical deficiencies, where restricted graft volume becomes a major limitation.

SPAL represents a different biologic model because its main conceptual strength is preservation of the periosteal vascular envelope as a protected augmentation compartment [6,12]. This approach is attractive for peri-implant buccal contour enhancement in selected esthetic regions [6,7,8,9]. However, current evidence remains limited, and predictability is likely influenced by periosteal plane control, defect morphology, tissue phenotype, restorative contour, and operator experience [6,12,13,14,20,21,22].

IDR occupies another conceptual category because its principal objective is immediate preservation or reconstruction of socket architecture in high-risk esthetic scenarios [10,11]. It is likely the most demanding of the reviewed techniques because extraction, implant placement, grafting, provisionalization, and contour control are compressed into one procedure. This explains why IDR remains highly operator-dependent and difficult to standardize. Importantly, much of the evidence frequently cited in support of IDR derives from broader immediate implant literature rather than protocol-specific IDR validation [18,19,23,24,25,26,27,28,29].

A major clinical implication of the proposed decision matrix is that minimal invasiveness may become biologic under-treatment when a limited intervention cannot provide sufficient regenerative volume, spatial stability, or soft-tissue support. Extensive three-dimensional ridge collapse, vertical defects greater than approximately 4–5 mm, combined horizontal and vertical deficiencies, lack of palatal or lingual support, severe phenotype-related esthetic risk, and inability to control implant three-dimensional position or primary stability are examples in which conventional staged augmentation may remain biologically superior despite higher surgical burden [1,2,7,8,9].

From a practical standpoint, minimally invasive regenerative approaches should be regarded as additions to the therapeutic armamentarium rather than replacements for established reconstructive methods. In limited defects and selected esthetic indications, they may reduce morbidity, shorten treatment time, and improve patient acceptance. In structurally demanding defects, conventional guided bone regeneration, staged augmentation, or block grafting may still provide greater reconstructive control and more reproducible outcomes. A serious limitation of the current field is the lack of standardized defect classification systems tailored to minimally invasive regenerative strategies.

Another critical issue is technique sensitivity and learning curve. Limited-access procedures may require greater precision because visual control is reduced and the margin for technical error is narrower. Outcomes depend on case selection, defect morphology, flap design, periosteal or socket-wall preservation, implant positioning, graft adaptation, provisionalization, restorative planning, and operator experience. These factors should be reported more consistently because they directly affect training requirements and interpretation of published outcomes.

This perspective also redefines how future comparative studies should be designed. Rather than comparing techniques under a broad less invasive versus more invasive paradigm, future research should stratify cases according to defect phenotype, esthetic risk, volume demand, soft-tissue thickness, implant timing, prosthetic design, and operator experience. Such indication-sensitive designs are more likely to determine whether reduced morbidity translates into comparable long-term hard- and soft-tissue stability [1,2,19,30,31,34].

A further clinically relevant implication concerns prosthetically driven contour support. In SPAL, the stability of the augmented buccal envelope may be influenced by abutment transition, crown contour, and loading-related soft-tissue support. In IDR, provisional restoration geometry and final emergence profile are integral to maintaining the reconstructed socket contour [7,8,9,30,31,34]. Future studies should therefore incorporate restorative variables, digital contour superimposition, and longitudinal volumetric monitoring into outcome reporting.

Overall, the currently available literature supports cautious and indication-specific optimism. Minimally invasive regenerative techniques should be viewed neither as replacements for established augmentation principles nor as simple procedural shortcuts. At present, the evidence supports their use in selected clinical scenarios, but remains insufficient to define them as universally applicable standards of care. Future comparative studies with standardized indications, longer follow-up, and integrated hard-tissue, soft-tissue, prosthetic, esthetic, and patient-centered outcomes are required to clarify their role in contemporary implant dentistry.

## 10. Conclusions

Minimally invasive bone regeneration represents an important conceptual evolution in implant dentistry, shifting attention from augmentation volume alone toward indication-specific preservation of local healing potential, vascular supply, socket architecture, and prosthetically relevant contour stability [1,5,6,10]. As highlighted by the conceptual framework proposed in Figure 9 and the indication-driven matrix in Table 5, the Bone Core Technique, SPAL, and IDR should not be interpreted as interchangeable less invasive alternatives, but rather as distinct strategies matched to different clinical scenarios and supported by different levels of evidence.

The currently available literature suggests that these approaches may provide favorable hard- and soft-tissue outcomes, reduced patient morbidity, and esthetic benefits only when applied under strict indication control and by experienced operators [1,5,6,8,9,10,12,21,30]. The strength of evidence remains uneven. The Bone Core Technique currently offers the strongest dedicated clinical follow-up for localized peri-implant defects, SPAL presents a promising periosteal-preserving model for buccal contour enhancement, and IDR remains an expert-driven immediate reconstruction concept with particular relevance in high-risk esthetic-zone sockets [5,6,10,11,12,13,14,15,16,17,20,21,22].

A key practical implication emerging from this review is that minimal invasiveness should be judged by biologic adequacy rather than by surgical size alone. In selected localized defects, tissue-preserving regenerative strategies may offer advantages. In contrast, extensive three-dimensional ridge collapse, severe vertical deficiencies, combined defects, and scenarios in which implant positioning, primary stability, or soft-tissue support cannot be predictably controlled may still be better managed through conventional staged augmentation procedures [1,2,7,8,9].

Accordingly, minimally invasive regenerative techniques should be regarded as indication-sensitive additions to the contemporary implant regenerative armamentarium rather than universal replacements for established GBR principles. Their long-term clinical value will depend on standardized defect classification, consistent reporting of buccal bone thickness, soft-tissue dimensions, volumetric contour changes, esthetic indices, implant positioning parameters, restorative variables, follow-up intervals, and prospective comparative studies capable of validating reproducibility across operators and clinical settings [1,2,19,30,31].

The proposed operational framework emphasizes that the key clinical question is not whether a procedure is less invasive, but whether it is biologically adequate for the specific defect. In this sense, minimally invasive treatment may be preferable for localized, contained, or socket-specific defects, whereas conventional staged reconstruction remains essential when the defect requires vertical support, three-dimensional space maintenance, or extensive hard- and soft-tissue rebuilding.

## 11. Future Directions

Future research in minimally invasive bone regeneration should move beyond technical feasibility and descriptive success toward clinically achievable comparative designs [1,2,19,30]. Prospective multicenter cohort studies and, where feasible, controlled comparative trials are needed to determine whether these protocols can achieve outcomes comparable with conventional augmentation procedures in terms of implant survival, peri-implant tissue stability, esthetic integration, complications, patient-reported morbidity, and treatment burden [1,2,19,30,31,34].

A major priority should be the standardization of outcome reporting [8,9,19,30,31]. Future studies should consistently characterize defect morphology, buccal bone thickness, soft-tissue dimensions, volumetric contour changes, esthetic indices, implant positioning parameters, restorative/provisionalization variables, operator experience, and follow-up intervals [8,9,19,30,31,34]. Without such standardization, meaningful comparison among studies and across techniques will remain limited.

Further investigation is also needed into the biologic mechanisms underlying these techniques [1,6,22]. Histologic and radiographic studies may help clarify the quality and maturation of regenerated tissues, the behavior of graft materials under periosteal-preserving or compartment-based conditions, and the long-term stability of reconstructed buccal contours [6,21,22]. In this respect, recent histologic observations related to SPAL are particularly relevant, but broader biologic validation remains necessary [21,22].

Another important direction for future research is the development of practical indication-specific treatment algorithms [1,5,6,10]. Not all peri-implant defects are equally suitable for minimally invasive management, and greater clarity is needed regarding the defect configurations, esthetic scenarios, prosthetic conditions, and patient-related factors that are most likely to benefit from these approaches [5,6,10,18,19]. Comparative frameworks should also specify when conventional staged reconstruction remains preferable [1,2].

An additional realistic priority is the integration of digital tools into diagnosis and follow-up. CBCT-based defect characterization, intraoral scan superimposition, digital volumetric contour monitoring, and guided implant positioning may improve case documentation and make outcome reporting more comparable. AI-supported decision tools are promising, but should be considered exploratory until validated against clinical outcomes and standardized datasets.

Finally, future publications should more explicitly address reproducibility, learning curve, and operator dependence [5,6,10,11,12,33,34]. Because many minimally invasive regenerative protocols are highly technique-sensitive, their broader adoption will depend not only on biologic plausibility but also on clinically reproducible workflows, training pathways, and transparent reporting of operator experience [5,6,10,12,33].

Considering recent evidence on the role of bone blocks in three-dimensional alveolar reconstruction [35], future investigations should clarify whether less invasive, defect-specific techniques can provide comparable regenerative stability with reduced patient morbidity and improved soft-tissue preservation in clearly defined indications.

At present, the most immediate research priority is not the introduction of more complex technologies alone, but the creation of standardized, reproducible, and clinically interpretable datasets that can support future digital and AI-assisted planning tools.

## Figures and Tables

**Table 1:** Technique-specific clinical thresholds, learning curve considerations, and potential complications.

Technique	Technical Threshold/Operator Requirements	Learning Curve Consideration	Potential Specific Complications
Bone Core Technique	Implant osteotomy control; atraumatic core harvesting; precise graft adaptation and fixation; magnification, core drills/trephines, fixation screws, and microsurgical retractors may be required.	No validated number of procedures is available; outcomes should be interpreted in relation to prior experience with implant placement, GBR, and small autogenous graft fixation.	Bone-core fracture or fragmentation; insufficient graft volume; overheating; fixation instability; flap trauma; residual peri-implant dehiscence.
SPAL	Controlled split-thickness/subperiosteal pouch preparation; preservation of periosteal thickness and vascularity; tension-free graft placement; microsurgical/tunnel instruments and magnification are desirable.	No validated threshold exists; reproducibility likely depends on experience with periosteal-plane surgery and limited-access augmentation.	Periosteal perforation; pouch collapse; graft migration; mucosal thinning; wound dehiscence; excessive flap tension; inadequate buccal contour gain.
IDR	Expert-level atraumatic extraction, immediate implant placement, tuberosity graft harvesting, socket reconstruction, provisionalization, and emergence-profile control.	No validated procedure number exists; favorable results from expert centers should not be generalized without documenting operator case volume and training.	Buccal wall collapse; loss of primary stability; graft displacement; mucosal recession; papilla loss; provisional overpressure; infection; esthetic failure.

**Table 2:** Key Features of the Bone Core Technique, SPAL, and IDR.

Technique	Main Indication	Biological Rationale	Surgical Invasiveness	Graft/Material Source	Main Advantages	Main Limitations	Current Evidence Profile
Bone Core Technique	Localized peri-implant bony defects, especially dehiscence-type defects at implant placement	Uses a locally harvested autogenous bone cylinder from the implant osteotomy; preserves autogenous biological potential while avoiding a second donor site	Low to moderate	Autogenous local bone core harvested during implant bed preparation	Single-field surgery; avoids second donor site; biologically attractive; reduced morbidity; suitable for simultaneous implant placement	Limited graft volume; highly dependent on defect morphology and surgical precision; not suited for large horizontal/vertical deficiencies	Best-supported of the three discussed techniques, including a prospective 5-year cohort study
SPAL	Buccal peri-implant dehiscence correction; contour enhancement where periosteal preservation is desirable; selected thin buccal plate scenarios	Preserves periosteal blood supply by minimizing flap elevation and using a subperiosteal space for augmentation	Low to moderate, but technically delicate	Usually xenograft-based, including particulate DBBM or bovine-derived block materials	Periosteal preservation; potential hard- and soft-tissue thickening; reduced flap-related trauma; attractive for buccal contour management	Technique-sensitive; limited comparative data; reproducibility outside experienced centers still unclear	Growing but still limited evidence: technical paper, case series, short-term tissue outcomes, peri-implantitis application, retrospective study, recent histologic case report
IDR	Highly selected immediate implant cases in the esthetic zone, especially compromised sockets with buccal wall loss/deficiency	Simultaneous reconstruction of the socket and peri-implant tissues at the time of immediate implant placement to preserve or restore ridge architecture	Moderate, despite the “minimally invasive” label, because several critical steps are compressed into one surgery	Commonly autogenous grafting, especially tuberosity-derived corticocancellous tissue/block in classic IDR descriptions	One-stage esthetic reconstruction; reduced total treatment time; immediate restoration of contour in selected cases; strong esthetic appeal	Very high technique sensitivity; operator dependence; difficult standardization; mostly case-based literature	Mainly conceptual papers, case reports/case series, and technique-driven literature; broader immediate implant reviews support feasibility but not IDR-specific superiority

Abbreviations: SPAL, Sub-Periosteal Peri-implant Augmented Layer; IDR, Immediate Dentoalveolar Restoration; DBBM, deproteinized bovine bone mineral.

**Table 3:** Current Evidence Base for Minimally Invasive Regenerative Techniques.

Technique	Key Reference(s)	Study Type	Follow-Up	Main Outcomes Reported	Key Limitations
Bone Core Technique	Khoury and Doliveux/Khoury et al. [5]	Prospective clinical cohort	≥5 years	Clinical and radiographic evaluation after simultaneous implant placement and augmentation of limited bony defects; favorable long-term performance in selected cases	No randomized comparison with conventional augmentation; technique-centered dataset; indication-restricted
SPAL	Trombelli et al., 2018. [6]	Technique article/case series	Short-term	Horizontal augmentation at implant placement; focus on periosteal preservation and coronal hard- and soft-tissue increase	Early-stage evidence; non-comparative
SPAL	Trombelli et al., 2019. [12]	Case series	Short-term	Simplified soft-tissue management for peri-implant bone augmentation	Small-scale, technique-driven evidence
SPAL	Trombelli et al., 2020. [13]	Retrospective case series	6 months after prosthetic loading	Peri-implant tissue conditions after SPAL; shallow probing depths and limited mucosal inflammation in treated implants	Short follow-up; no direct comparator
SPAL	Trombelli et al., 2020. [14]	Case report/technical application	Case-based follow-up	Use of SPAL in peri-implantitis lesions	Very low evidence level; non-generalizable
SPAL	Severi et al., 2025. [21]	Retrospective study	Mid-term	Correction of peri-implant buccal bone dehiscence using SPAL with either block or particulate xenograft; clinically relevant buccal augmentation outcomes	Retrospective design; still limited external validation
SPAL	Severi et al., 2025. [22]	Histologic case report	Case-based	Histologic characterization of newly formed tissue after SPAL plus particulate xenograft	Histology from isolated case context; cannot establish clinical predictability
IDR	da Rosa et al., 2013. [10]	Technique paper/case-based report	Clinical follow-up within case protocol	Introduced IDR as a one-stage approach for compromised sockets using autogenous grafting	Foundational but not comparative; largely conceptual
IDR	da Rosa et al., 2014. [15]	Clinical case report	Case-based	Immediate implant placement, reconstruction of compromised sockets, and recession repair with triple graft from tuberosity	Case report; high operator dependence
IDR	Franceschi et al., 2018. [16]	Clinical case report	Case-based	Application of IDR with maxillary tuberosity graft to reconstruct buccal wall loss	Single-case evidence
IDR	da Rosa et al., 2019. [17]	Technique-focused clinical article	Case-based/short-term	Combined use of IDR and osseodensification in immediate implant rehabilitation	Limited generalizability
Immediate implant background relevant to IDR	Ickroth et al., 2025; Riachi et al., 2024; Slagter et al., 2014. [18,19,28]	Systematic reviews/meta-analyses	Variable	Suggests favorable immediate implant outcomes in selected esthetic-zone cases, but protocols are heterogeneous and evidence in non-intact sockets remains limited	These data support the biologic context of IDR, but should not be interpreted as IDR-specific superiority

**Table 4:** Level of Evidence, Clinical Promise, and Main Limitations of Minimally Invasive Regenerative Techniques.

Technique	Highest Practical Evidence Level in This Review	Overall Clinical Promise	Main Limitation	What Still Requires Validation
Bone Core Technique	Prospective cohort with long-term follow-up	Promising for localized defects when limited autogenous augmentation is sufficient	Restricted graft volume and narrow indication range	Comparative effectiveness versus conventional augmentation and reproducibility across centers
SPAL	Technical reports, retrospective data, and early histologic support	Promising biologic concept for periosteal-preserving buccal contour augmentation	Limited comparative evidence and operator dependence	Long-term stability, external reproducibility, and indication boundaries
IDR	Predominantly case reports, case series, and concept-driven literature	May offer benefits in highly selected esthetic-zone immediate implant cases	Very high technique sensitivity and limited protocol standardization	Prospective comparative data, patient-reported outcomes, and broader generalizability

**Table 5:** Indication-Driven Clinical Decision Matrix for Minimally Invasive Bone Regeneration in Implant Dentistry.

Clinical Variable	Bone Core Technique	SPAL	IDR	Conventional GBR/Staged Reconstruction
Defect morphology	Localized peri-implant dehiscence or contained limited bony defect	Buccal peri-implant dehiscence, contour deficiency, shallow horizontal buccal loss	Compromised immediate socket in esthetic zone, mainly buccal wall deficiency	Extensive horizontal and/or vertical ridge deficiency
Primary biologic objective	Local autogenous osteogenic support	Periosteal vascular preservation and buccal contour thickening	Immediate dentoalveolar architecture preservation	Volume reconstruction with maximal spatial control
Implant timing	Simultaneous with implant placement	Simultaneous with implant placement	Immediate implant placement only	Early or delayed, often staged
Soft-tissue phenotype importance	Moderate	High	Very high	Moderate to high
Need for contour overbuilding	Low	Moderate to high	High	High
Volume requirement	Very limited	Limited to moderate	Moderate (socket-related)	Moderate to extensive
Best esthetic indication	Localized peri-implant buccal defect	Buccal contour enhancement in visible zone	High-risk anterior maxilla immediate implant case	Severe esthetic ridge collapse requiring staged rebuild
Technique sensitivity	High	Very high	Extremely high	Moderate to high
Dependence on operator expertise	High	Very high	Extremely high	High
Patient morbidity advantage	High	High	Moderate to high	Lower
Where NOT preferred	Large horizontal/vertical defects	Thin phenotype with poor periosteal control or extensive 3D collapse	Inability to obtain ideal 3D implant position, low primary stability, uncontrolled soft tissue	Small contained defects where biologic preservation is sufficient
Current evidence strength	Moderate	Low to moderate	Low	High
Best clinical role	Targeted biologic micro-augmentation	Periosteal-preserving contour management	Expert-driven esthetic rescue in immediate sockets	Gold standard for major reconstructive needs

## Data Availability

No new data were created or analyzed in this study. Data sharing is not applicable to this article.

## Abbreviations

The following abbreviations are used in this manuscript:

SPAL	Sub-Periosteal Peri-implant Augmented Layer
IDR	Immediate Dentoalveolar Restoration
DBBM	deproteinized bovine bone mineral
CBCT	Cone-beam computed tomography
